# Supplementary material for: Heterogeneous tumor blood oxygenation dynamics during phototherapy deciphered with real-time label-free photoacoustic imaging
Source: NPJ Acoust. 2025 Jun 4;1(1):9. doi: 10.1038/s44384-025-00012-x (PMC12137135; doi:10.1038/s44384-025-00012-x)
Supplement: Supplementary file 1 — Supplementary_Description_Table [file 44384_2025_12_MOESM1_ESM.docx]

**Supplementary**

| Video 1 | Movie of all PA StO_2_ images captured pre, during, and post-PDT of the mouse tumor featured in Fig. 3, along with associated animated graph from Fig. 3a. |
| --- | --- |
| Video 2 | Movie of all PA StO_2_ images captured pre, during, and post-PDT of the mouse tumor featured in Fig. 6, along with associated animated graph from Fig. 6b. |
